# Nintedanib reduces severity of post-traumatic joint contracture by modulating fibrosis and inflammation

**DOI:** 10.1007/s00109-025-02593-2

**Published:** 2025-09-25

**Authors:** Erik Wegner, Dennis Warnke, Victoria Buschmann, Benedikt Hild, Berenika Mais, Ulrike Ritz, Austin Harper, Erol Gercek, Philipp Drees, Andreas Baranowski

**Affiliations:** 1 Department of Orthopaedics and Traumatology, Biomatics Group, University Medical Center of the Johannes Gutenberg University, 55131 Mainz, Germany; 2 St. George’s University School of Medicine, True Blue, St. George, Grenada; 3 Department of Trauma Surgery, Orthopaedics and Reconstructive Surgery, ANregiomed Hospital, 91522 Ansbach, Germany

**Keywords:** Nintedanib, Myofibroblast, Fibrosis, PTJC, Translational medicine

## Abstract

**Abstract:**

This study investigates the potential of nintedanib, a tyrosine kinase inhibitor with antifibrotic and anti-inflammatory properties, to mitigate post-traumatic joint contracture (PTJC) in a rat model. Given the lack of effective pharmacological treatments for this debilitating condition, this study aims to address the unmet need for non-surgical interventions by targeting the underlying fibrotic and inflammatory processes. A total of 26 male Sprague–Dawley rats were subjected to standardized knee trauma and immobilization for 2 weeks. Rats were randomized into two groups: a nintedanib treatment group (5 mg/kg taken twice daily, *n* = 13) and a placebo group (*n* = 13). Joint mobility was evaluated biomechanically by measuring the contracture angle (CA) and resistance to extension. Posterior joint capsule tissues were analyzed histologically and via qPCR for profibrotic gene expression, including *α-Sma*, *Il-6*, *Tgf-β1*, *Nf-κb*, and *Ctgf*. Nintedanib treatment significantly reduced CA compared to placebo (68.1° ± 12.6° vs. 84.8° ± 11.1°, *p* < 0.01), indicating improved joint mobility. Knee extension in the nintedanib-treated rats required less force, particularly at lower extension angles (*p* < 0.05). Molecular analysis showed a marked reduction in *α-Sma* expression, a myofibroblast marker, in the nintedanib group compared to placebo (11-fold decrease, *p* < 0.05). Histological examinations revealed relatively fewer myofibroblasts in the posterior joint capsule of rats treated with nintedanib. Nintedanib effectively mitigates fibrosis and inflammation in a rat model of PTJC, enhancing joint mobility and reducing profibrotic gene expression. These findings support further exploration of nintedanib as a pharmacological therapy for PTJC in clinical settings.

**Key messages:**

- Nintedanib is a promising candidate for the prevention of post-traumatic joint contracture.
- Oral administration of nintedanib (5 mg twice daily) over a period of 2 weeks improves joint mobility in post-traumatic joint contracture (PTJC).
- Nintedanib reduces the relative number of myofibroblasts.
- A significant reduction in *α-SMA* expression levels under the influence of nintedanib indicates a slowed transition of fibroblasts to myofibroblasts.

## Background

Post-traumatic joint contracture (PTJC) is a common complication following trauma or injury to a joint, most frequently affecting the large joints. It is characterized by a restricted range of motion in the affected joint, which can profoundly impair an individual’s quality of life. After an injury, the joint capsule typically becomes thickened and contracted, a process associated with an increase in myofibroblasts and excessive extracellular matrix production [1–3]. Several factors can contribute to an increased risk of PTJC, such as younger age, reduced muscle strength, high-energy trauma, prolonged immobilization, multiple surgeries, and alcohol abuse [4]. This condition can significantly diminish quality of life; for instance, a stiff knee can impede an individual’s ability to ambulate. Moreover, contracture is often accompanied by persistent pain, further restricting mobility and significantly affecting overall well-being. Additionally, the inability to perform daily tasks can lead to psychological distress, such as frustration, decreased independence, and, in some cases, depression. Depending on the joint involved and the severity of the contracture, one’s capacity to work or engage in recreational activities may be affected. If left untreated, post-traumatic joint contracture can result in chronic dysfunction, potentially necessitating surgical intervention [5, 6].

Treatment for post-traumatic joint contracture generally involves physical therapy, bracing, and, in certain cases, surgery. Early detection and timely intervention are essential for minimizing its impact on quality of life and enhancing patient outcomes. While pharmacological agents such as ketotifen, montelukast, glucocorticoids, and celecoxib have shown promise in improving joint contracture in animal studies, their clinical application remains limited [7–10]. This underscores the ongoing need for exploring and screening potential non-surgical pharmacological therapies.

**Fig. 1:**
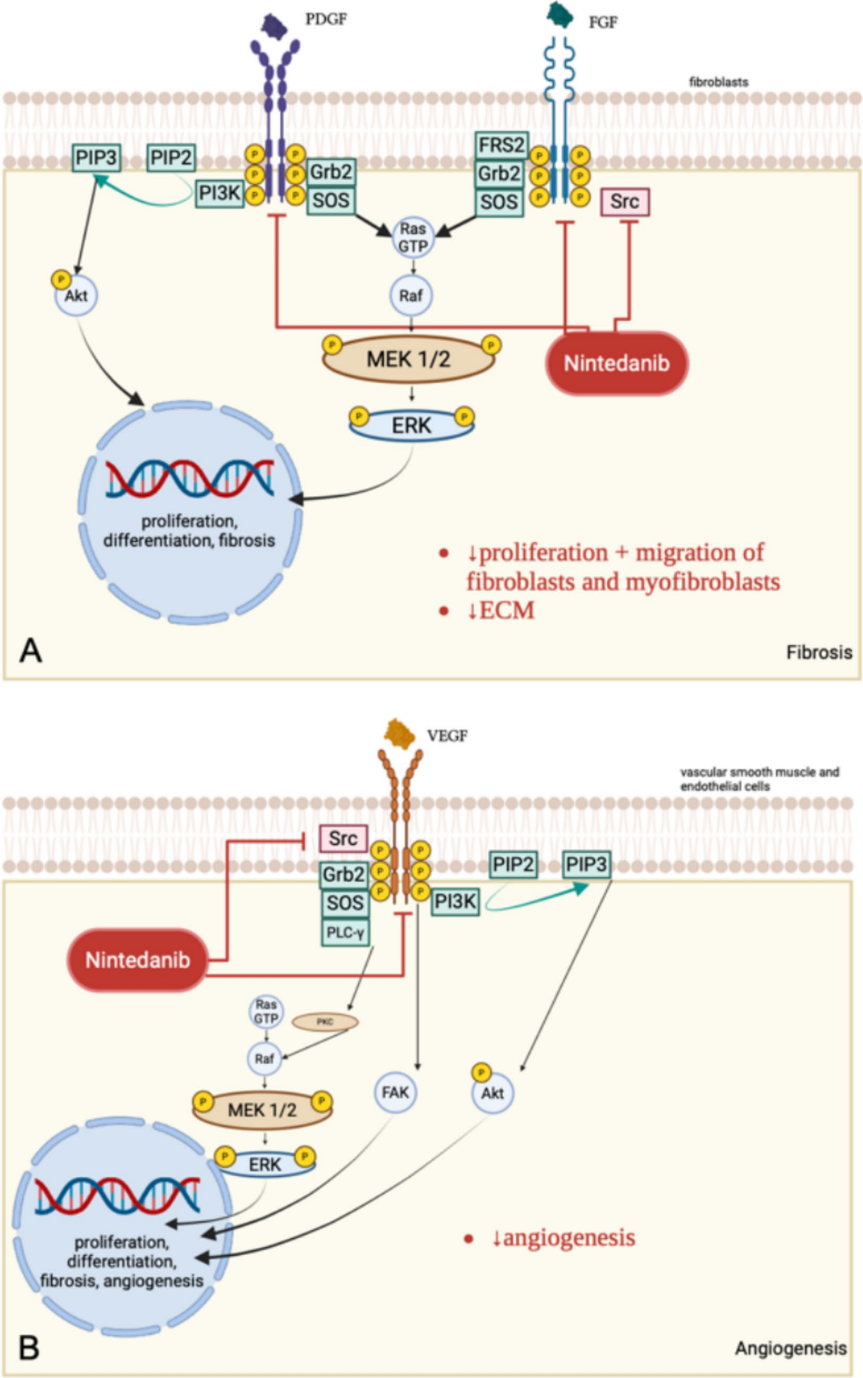
Mode of action of the small molecule tyrosine kinase inhibitor nintedanib. **A** Nintedanib binds to the platelet-derived growth factor receptor (PDGFR) and the fibroblast growth factor receptor (FGFR) on fibroblasts via intracellular ATP-binding pockets and prevents their autophosphorylation. In addition, it inhibits the profibrotic downstream signaling cascades via the non-receptor tyrosine kinase Src. Profibrotic influence of VEGF via PDGFR is not demonstrated. **B** Nintedanib prevents angiogenesis by binding intracellularly to the ATP-binding pocket of the vascular endothelial growth factor receptor (VEGFR)-2 and by inhibiting SRC [15, 17] (created in https://BioRender.com)

Although not specifically studied for post-traumatic joint contracture, nintedanib holds considerable promise in targeting the underlying mechanisms of this condition. As a potent tyrosine kinase inhibitor with powerful antifibrotic properties, it has proven effective in reducing pulmonary fibrosis in models of rheumatoid arthritis-associated interstitial lung disease [11, 12]. Rheumatoid arthritis and arthrofibrosis share notable similarities in their pathogenesis. In fibroblast subcluster analysis, both diseases exhibited an increased number of the pathogenic cell types CD34 + sublining fibroblasts (CD34-SLF) and DKK3 + sublining fibroblasts (DKK3-SLF). These cells produce excessive cytokines and chemokines, such as IL-6 and CCL2, and may contribute to the progression of arthrofibrosis through elevated secretion of TGF-β and PDGF. Nintedanib could potentially offer a targeted intervention, like its role in the treatment of rheumatoid arthritis-associated interstitial lung disease, and mitigate the development of arthrofibrosis [13]. Its antifibrotic properties may stem from the suppression of mRNA expression for extracellular matrix (ECM) in joint capsules. Since excessive ECM deposition is a critical factor in joint contracture, this reduction could help prevent or alleviate contracture [3, 11]. Additionally, Nintedanib effectively inhibits the activity of key receptor tyrosine kinases (RTKs), including fibroblast growth factor receptors (FGFRs) and platelet-derived growth factor receptors (PDGFRs). By blocking FGFR and PDGFR signaling pathways, nintedanib interferes with critical processes such as fibroblast activation, proliferation, and extracellular matrix deposition (Fig. 1). This disruption might play a role in slowing the progression of post-traumatic joint contracture [14, 15]. In addition to its antifibrotic effects, nintedanib has also exhibited anti-inflammatory properties [12]. Inflammation plays a significant role in the development of post-traumatic joint contracture [7, 16]. Regulating pro-inflammatory cytokines may inhibit the recruitment of myofibroblasts, which are believed to be the primary effector cells in contracture progression [16]. An in vivo study by Redente et al. demonstrated that early intervention with nintedanib in mice significantly reduced arthritis development [11]. This suggests that early administration of nintedanib following joint trauma could potentially prevent the onset of contracture.

In summary, post-traumatic joint contracture remains a significant clinical challenge that, if left untreated, can severely impair function and quality of life. While conventional treatments focus on rehabilitation and surgery, the exploration of pharmacological agents like nintedanib offers promising new avenues for preventing and treating the condition. To assess whether the aforementioned effects of nintedanib can also be transferred to post-traumatic joint contracture, we conducted a randomized placebo-controlled trial of nintedanib administration after knee injury in a rat model. The primary endpoint of this study was to determine whether the administration of nintedanib was associated with improved knee joint mobility in our animal model of post-traumatic joint contracture. In addition, the inhibitory effect of the drug on joint capsule fibrosis and pro-inflammatory cytokines and their effector genes was investigated.

Our findings will help to provide a preventive drug treatment for post-traumatic joint contracture.

## Methods

### Study design

**Fig. 2:**
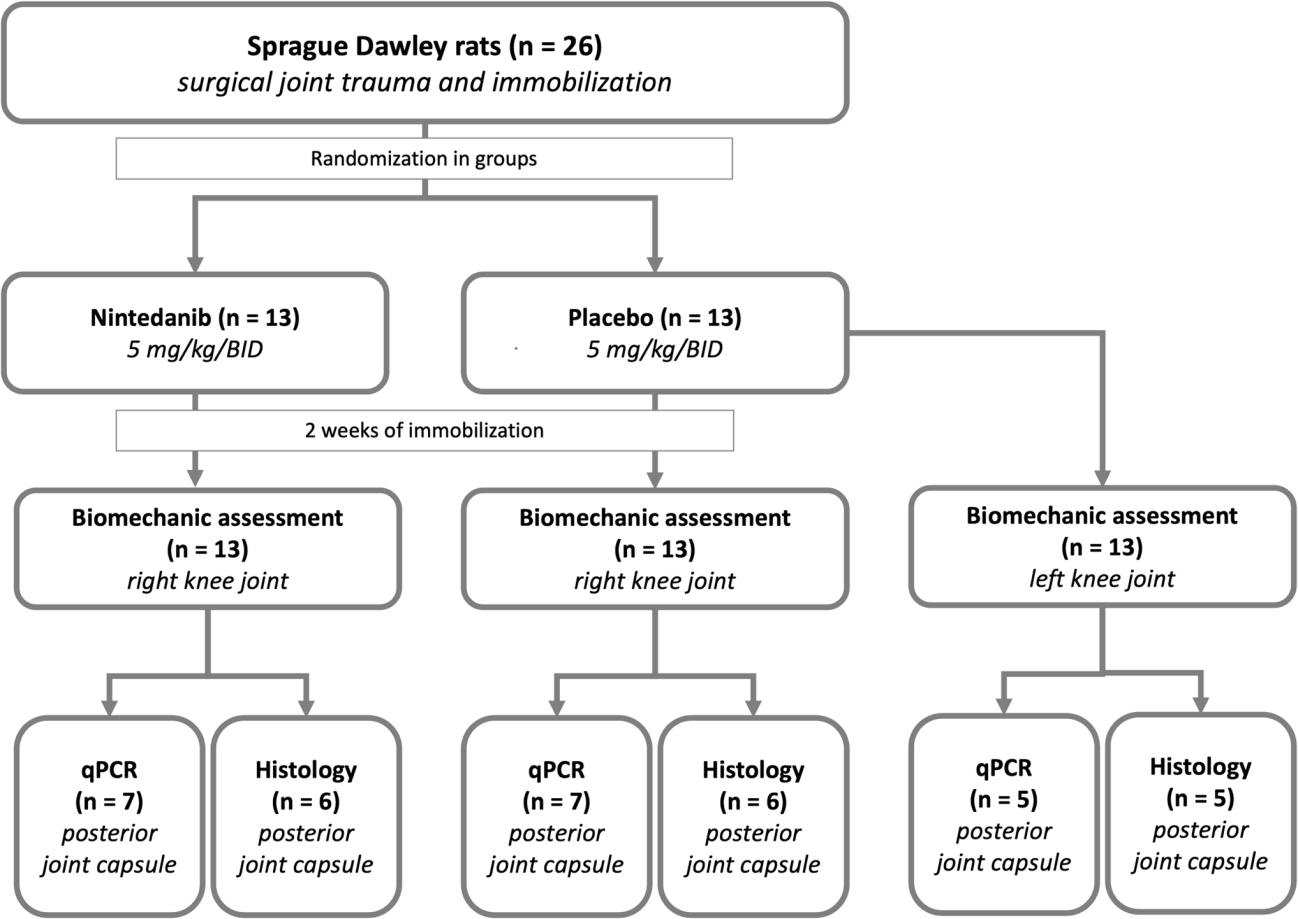
Study design. Allocation of animals to nintedanib or placebo groups. All animals (*n* = 26) underwent the same surgical trauma and immobilization of the right knee joints. The animals were then randomized to the nintedanib group (*n* = 13) or the placebo group (*n* = 13). After 2 weeks of immobilization, a biomechanical analysis was performed, and the posterior joint capsule, after re-randomization, was used for either histological (*n* = 6) or gene expression analysis (*n* = 7). The uninjured left legs (*n* = 13) of the placebo group were also analyzed, and their range of motion was used as a reference angle for the physiological mobility of uninjured rat knees. Bis in die (BID)

The experiments were conducted using male Sprague–Dawley rats, aged 11 weeks (*n* = 26) (Janvier Labs, Saint-Berthevin Cedex, France), with an average weight of 447.3 ± 27.9 g. Each rat was individually housed under standard conditions, with a 12:12 h light–dark cycle at room temperature. They were provided with commercial rodent chow and water ad libitum. The animals were divided into two groups: a nintedanib treatment group (*n* = 13) and a placebo group (*n* = 13). The sample size was calculated in collaboration with the Institute of Medical Biometrics, Epidemiology, and Computer Science at the University of Mainz, using a significance level of *α* = 5% and a power of *β* = 80%. Randomization of the groups was completed using the “Random numbers calculator” provided by GraphPad (https://www.graphpad.com/quickcalcs/randmenu/) before surgery. On day 0, both groups underwent a standardized procedure that involved creating an injury to the posterior capsule of the right knee joint, followed by surgical immobilization of the joint using a Kirschner wire (K-wire). The left knee of the placebo group was used as a control. From the first day of immobilization until euthanasia, the rats received nintedanib esylate (5 mg doses twice per day, orally; manufactured by Boehringer Ingelheim International GmbH, Ingelheim am Rhein, Germany) or a placebo (5 mg doses twice per day, orally; P-Tabletten Weiss, manufactured by Winthrop Arzneimittel GmbH, Frankfurt am Main, Germany) at 12-h dosing intervals (Fig. 2). The placebo consisted of lactose monohydrate, cellulose powder, magnesium stearate, and microcrystalline cellulose. The drugs were ground in a mortar and mixed with white chocolate cream (0.25 g/dose), which was then offered to the animals. The animals preferred the treat to their normal food and ate it immediately.

No comparative data on nintedanib dosage in a rodent model of arthrofibrosis currently exist. The chosen dosage is based on the no-observed-adverse-effect level (NOAEL) identified from existing literature, as well as its demonstrated antifibrotic effect in the bleomycin-induced lung fibrosis model in rats [15, 18]. After 2 weeks of immobilization, all animals were euthanized. Euthanasia was performed via CO_2_ asphyxiation. The exclusion criteria for animals were defined in advance. These included a body weight loss of > 20% and abnormal behavior, such as apathy and self-mutilation. Material failure, including K-wire dislocation and fracture of the material or the femur or tibia, as well as wound healing disorders or wound infections, also led to the exclusion of the experimental animals. All experimental procedures were conducted in a double-blinded manner, and the study received approval from the relevant ethics committee (ID 23177–07/G 21–1-113).

### Rat model and operative technique

**Fig. 3:**
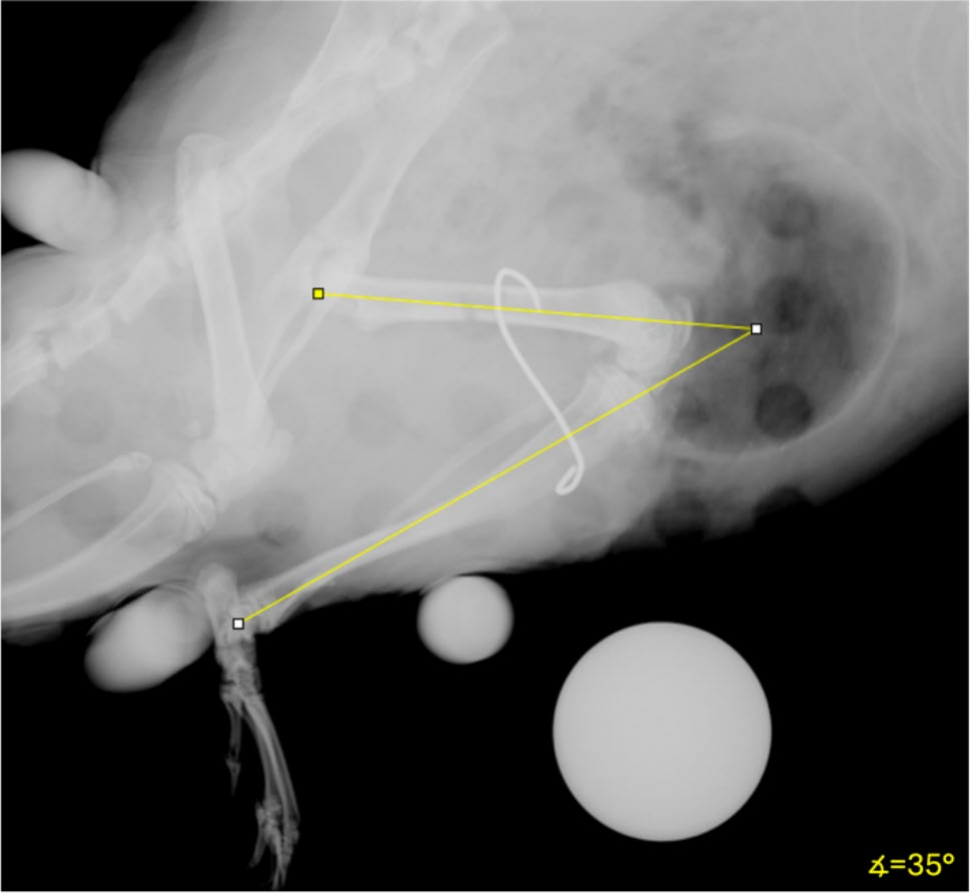
Lateral X-ray of the knee joint. A joint angle of 35° was fixed using a K-wire arthrodesis

**Fig. 4:**
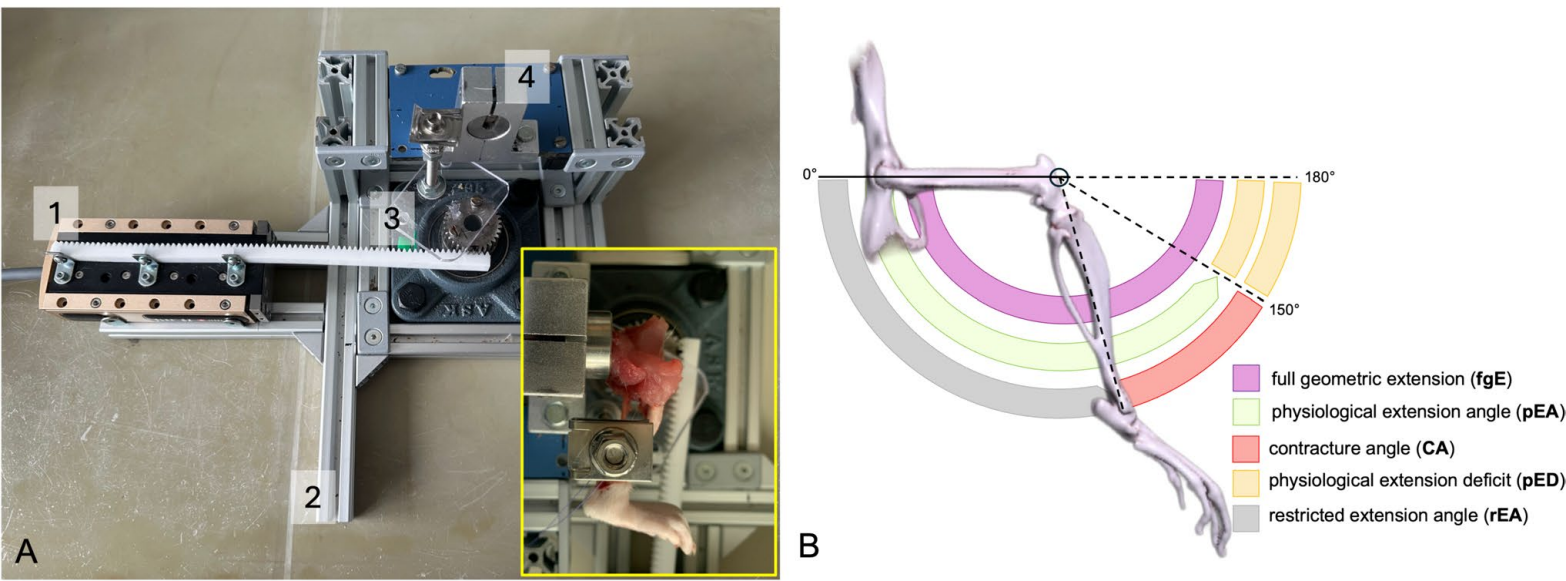
**A** Orthogonal view of the automated arthrometer. (1) Linear motor slide with straight gear rack, (2) aluminum frame, (3) gear transmission with mobile stop clamp, and 4 static stop clamp. The Servo Controller is not shown. The inset image shows a loaded sample. **B** 3D simulation based on CT of the lower extremity of a rat. The inner circle depicts full geometric extension, the middle circle a physiological ROM, and the outer circle a pathological ROM after post-traumatic joint contracture. The full geometric extension is limited by the physiological extension deficit (orange). An additional contracture (red) further reduces the range of motion

Joint trauma was induced according to our standard protocol, as previously detailed [19]. The rat model utilized in this study involved the induction of a posterior joint capsule rupture, hemarthrosis, intra-articular bone damage, and temporary joint fixation. The surgical procedure was performed on the right limb of each animal under sterile conditions. In detail, the posterior joint capsule was traumatized by a joint angle of 180° hyperextension (Fig. 4B). A stab incision was then made over the anteromedial lower leg. The patellar ligament was exposed. A 1.0-mm-thick K-wire was inserted through the patellar ligament. A precise hole, 1.0 mm in thickness and 3.0 mm in depth, was drilled into the intercondylar region, with careful preservation of the weight-bearing cartilage and articular ligaments. An X-ray (Faxitron MX-20 Cabinet X-Ray System) in two planes was used to confirm the correct position of the K-wire and to rule out periarticular fractures. A lateral approach to the femur was chosen for the temporary K-wire arthrodesis. This was also performed via a stab incision. The thigh fascia was opened along the course of the femoral muscles. This was followed by anterior retraction of the vastus lateralis muscle to expose the femoral shaft. The tibia was exposed using the approach mentioned at the beginning. An anterior–posterior 1.2 mm diameter, 15° angled hole was drilled in the middle third of the tibia and in the middle third of the femur. A blunt 0.6 mm diameter K-wire was inserted through the tibial drill hole, the periarticular soft tissue, and finally through the femoral drill hole. The K-wire was bent into a hook shape around the femur and then pulled back until a joint angle of 35° was achieved. The flexion angle and the K-wire position of the arthrodesis were verified by a lateral X-ray (Fig. 3). The tibial end of the K-wire was bent over the tibia to prevent any joint movement. Then, the wound was closed in layers. After 2 weeks of immobilization of the knee joint, the implant failure was again ruled out radiologically, and the K-wire was removed through the original surgical approach.

All surgical procedures were carried out under general anesthesia. Anesthesia was induced with 1% isoflurane inhalation and then maintained with subcutaneous injections of 0.005 mg/kg fentanyl, 4.0 mg/kg midazolam, and 0.375 mg/kg medetomidine. After the surgery, general anesthesia was reversed using flumazenil (0.2 mg/kg) and atipamezole (1 mg/kg). For postoperative analgesia, tramadol (1 mg/mL) was added to the drinking water, beginning 3 days before surgery and continuing for 7 days postoperatively. To mitigate the potential impact of muscle tension on joint contracture, periarticular myotomy was performed prior to all joint angle measurements. This procedure involved dissecting the skin and cutting all surrounding soft tissues 10 mm above and below the joint line.

### Joint angle measurements

The joint angle refers to the angle between the femur and tibia. If the femur and tibia align in a straight axis, the extension angle is 180° and the knee joint is fully extended. Since rats cannot achieve a 180° extension angle, the difference between the maximum extension angle of a healthy rat knee at a torque of 35 Nmm and the full extension (180°) was defined as the “physiological extension deficit (pED)” (Fig. 4B). Therefore, a healthy rat knee, by definition, does not exhibit contracture (i.e., a pathological restriction of movement). A torque of 35 Nmm has been shown to result in complete physiological extension in healthy knee joints without causing structural damage to the internal structures of the knee [7, 19, 20]. Joint angle measurements were performed in both the nintedanib group and the placebo group. Since the operations and immobilizations were performed on the right knee joints, the left knees of the placebo group served as healthy controls (Fig. 2). The extent of knee joint contracture was calculated by determining the difference between the mean extension angle of the left knee (control) and the measured extension angle of the corresponding right knee joint (Fig. 4B). All joint angle measurements were performed on the rats’ legs immediately after euthanasia, 2 weeks after the initial surgery. To assess the pathological contracture of the joint capsule, i.e. the arthrogenic component of the contracture, the skin and periarticular muscles were completely severed 1 cm from the femoral and tibial joint line prior to the measurements.

**Fig. 5:**
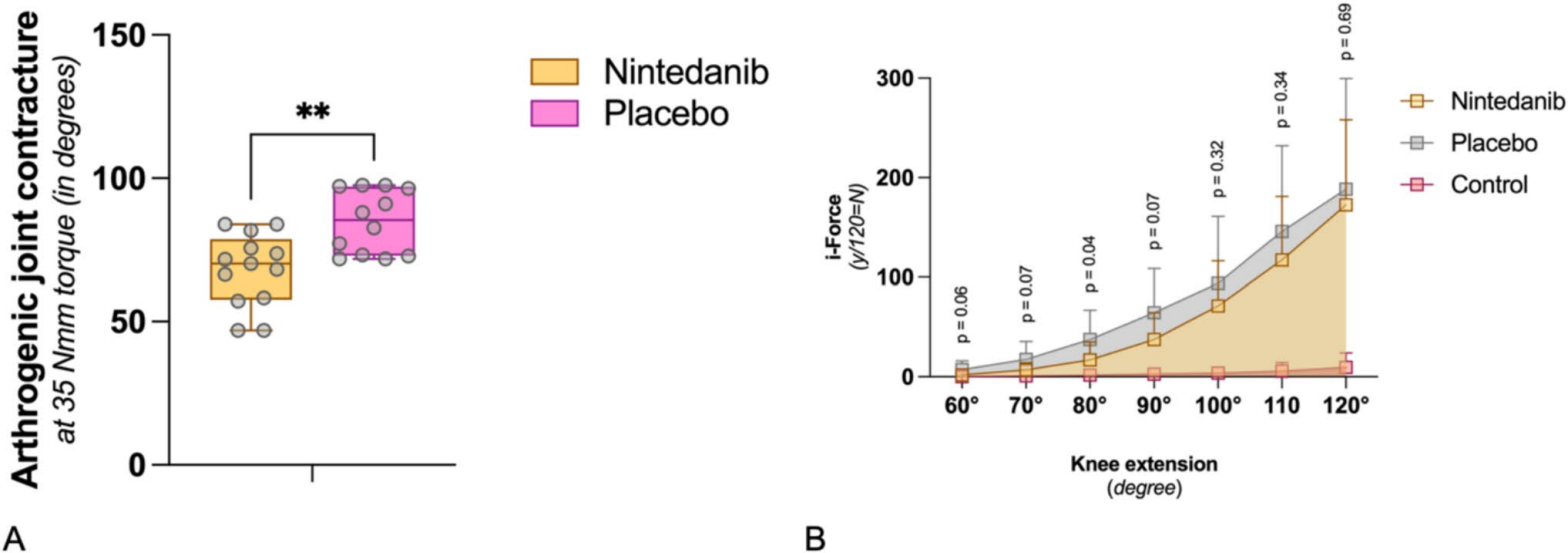
Biomechanical evaluation. **A** The graph compares the arthrogenic joint contracture with nintedanib (*n* = 13) or placebo (*n* = 12) at 35 Nmm torque after immobilization for 2 weeks. Median, maximum, and minimum values are presented. Statistically significant differences (Mann-Whitney *U*-Test) between groups are marked with ** (*p* ≤ 0.01). **B** The graphic compares the arthrogenic joint contracture dynamically following the administration of nintedanib (*n* = 13) or placebo (*n* = 12) after 2 weeks of immobilization. The *p*-values shown are a comparison of the area under the curve between 60° extension angle and the angle of the specified *p*-value. In addition, the healthy knee joints (control) are plotted (*n* = 13). I-Force is the product of the used amperage and a device-specific constant (1*N* = 12*10 mA). Median, maximum, and minimum values are presented. *P*-values (multiple *t*-test with Welch correction) are given above for the specific endpoint of the extension angle. To better illustrate the significance at small joint angles, values above 120° have been omitted from the graph

A custom-built automated mechanical testing system was created to evaluate joint contracture in rat knees (Fig. 4A), modeled after systems previously used for testing rabbit knees and rat elbows [21–23]. The system incorporates a linear motor slide (ELAX Ex 50F20, Jenny Science AG, Rain, CH), coupled with an actuator that provides linear displacement and measures force using Forceteq force–displacement technology (Jenny Science AG, Rain, CH), all controlled via the servo controller (XENAX Xvi Servo Controller with SMU (Safety Motion Unit) SS2 (Safe Stop 2), Jenny Science AG, Rain, CH). Linear displacement is converted into rotational motion via a rack-and-pinion gear, allowing for load-controlled testing of the rat knee joint in flexion–extension. After securing the limb stop clamps, a single load cycle was performed. The cycle consisted of extending the joint from its starting position to an extension angle of 180°. Force and torque were measured continuously throughout the cycle. Force–displacement data were recorded using Forceteq technology. The datasets were converted to torque and angular position. The angular position of the knee was calculated from the stroke position of the linear motor and transmitted to the program. Joint contracture was assessed by measuring maximum extension. The static and dynamic evaluation of the contracture was calculated from the same load cycle (Fig. 5). For the static evaluation, the joint angle was determined when a torque of 35 Nmm was reached in the load cycle.

### Tissue preparation for histological analysis

For histological analysis, the knee joints were carefully dissected immediately following euthanasia (*n* = 6 in the nintedanib group, *n* = 6 in the placebo group, and *n* = 5 in the unoperated control/left knee). The tissues were subsequently preserved in neutral buffered formalin solution (4.5%) (Carl Roth, Karlsruhe, Germany) for a duration of 48 h. Decalcification was carried out thereafter using a tris(hydroxymethyl)aminomethane (TRIS)-buffered 17.7% ethylenediaminetetraacetic acid (EDTA) solution (AppliChem, Darmstadt, Germany) for 6 weeks at 20 °C temperature, with continuous agitation by a roller mixer. The tissue samples were then embedded in paraffin, and sagittal Sects. (5 µm thick) were obtained from the central region of the knee joint. Any sections showing tissue-specific artifacts or structural damage to the meniscus or adjacent capsule structures were excluded. For morphometric quality evaluation, the sections were stained with hematoxylin and eosin (H&E) following standard procedures [24].

**Fig. 6:**
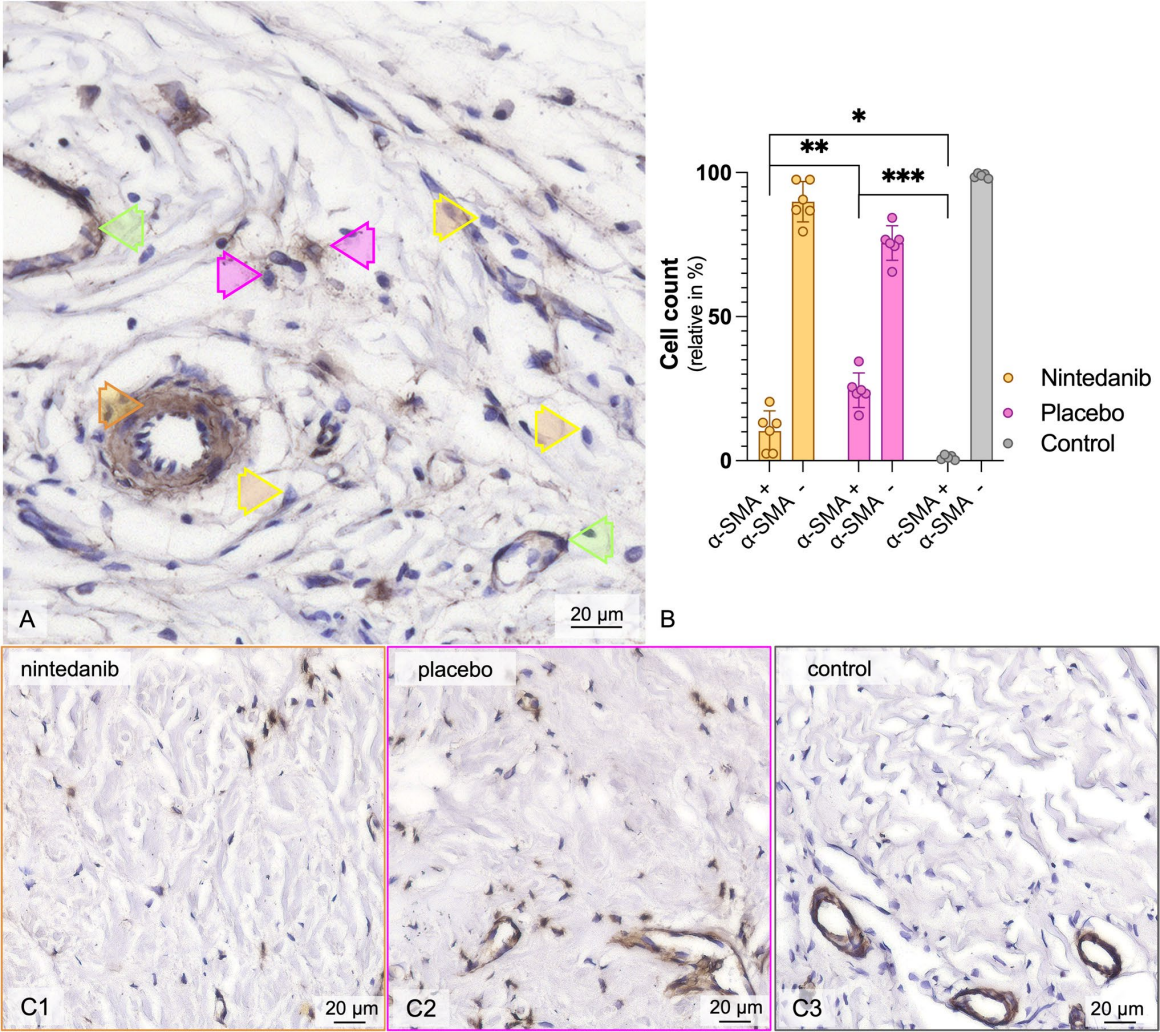
**A** α-SMA staining posterior joint capsule. A section of the posterior joint capsule stained with *α-Sma* and hematoxylin of a nintedanib treated rat is depicted. Pink arrows denote *α-Sma*( +) myofibroblasts, while yellow arrows indicate *α-Sma*( −) fibroblasts. Additionally, in close proximity to blood vessels, a green arrow identifies an *α-Sma*( +) pericyte, and an orange arrow points to *α-Sma*( −) smooth muscle cell. **B** Bar chart of the relative number of *α-Sma*( +) and *α-Sma*( −) cells in an HPF. Nintedanib significantly reduces the myofibroblast count. Significant differences are marked either with * (*p* ≤ 0.05), ** (*p* ≤ 0.01), or *** (*p* ≤ 0.001, one-way ANOVA and post hoc Tukey multiple comparison test). **C1–3** Examples of histological sections from the **C1** nintedanib, **C2** placebo, and **C3** control groups

Images of the tissue sections were captured and analyzed using ImageJ (v. 1.54 m). For cell quantification, an area measuring 62,500 µm^2^ (250 × 250 µm), referred to as the staining area or high-power field (HPF), adjacent to the posterior margin of the meniscus was determined. Evaluation was performed by two independent investigators who were blinded to the group assignments. For myofibroblast quantification, immunohistochemical *α-Sma* staining was performed. The staining was performed as described in our preliminary work [24]. To account for variability in tissue cellularity and sectioning, *α-Sma* + myofibroblasts were quantified as a proportion of total cells. For each specimen, nine high-power fields (across three slides) were analyzed, and *α-Sma* + cells were expressed as the percentage of total cells per HPF; the nine field proportions were averaged. This normalization to total cell count minimizes bias from differences in cellular density and staining efficiency across samples. Cell differentiation of *α-Sma* positive ( +) cells was assessed in relation to their proximity to a vascular lumen. *α-Sma* ( +) cells in the vicinity of vascular structures were excluded as pericytes or smooth muscle cells [25, 26]. All other *α-Sma* ( +) cells were defined as myofibroblasts. *α-Sma* negative ( −) cells located in the ECM were classified as fibroblasts (Fig. 6).

### Tissue preparation for quantitative PCR

The posterior section of the knee joint capsule (*n* = 7 in the nintedanib group, *n* = 7 in the placebo group, and *n* = 5 in the unoperated control/left knee) was immediately excised after euthanasia and immersed in RNAlater (Thermo Fisher Scientific, Waltham, USA). It was stored at – 20 °C until further processing for quantitative polymerase chain reaction (qPCR): ribonucleic acid (RNA) was isolated by manually grinding the samples in liquid nitrogen, followed by additional homogenization using a Precellys homogenizer (Bertin Technologies, Montigny-le-Bretonneux, France) in a TRIzol suspension (Thermo Fisher Scientific), in line with the manufacturer’s instructions. The supernatants from the centrifuged homogenates were processed using a standard phenol–chloroform RNA extraction protocol (Sigma-Aldrich, St. Louis, USA). RNA samples were suspended in nuclease-free water (Sigma-Aldrich) and quantified photometrically at 260 nm with a NanoDrop spectrophotometer (Thermo Fisher Scientific). Subsequently, 0.8 µg of RNA per sample was reverse-transcribed into complementary DNA (cDNA) using M-MuLV reverse transcriptase, Random Primer Mix (New England Biolabs, Ipswich, USA), and nucleotides (Bioron GmbH, Ludwigshafen, Germany). The primers for qPCR (Table 1) were designed based on nucleotide sequences provided by the National Center for Biotechnology Information (https://www.ncbi.nlm.nih.gov/nucleotide/), using a web-based primer design tool from the manufacturer (Eurofins Scientific, Luxembourg City, Luxembourg). The qPCR reactions were carried out on the qTOWER3 system (Jena Analytik, Jena, Germany) with Blue S’Green qPCR Master Mix (Biozyme Scientific GmbH, Hessisch Oldendorf, Germany) in accordance with the manufacturer’s instructions. ΔCt (Δ-cycle threshold) values were used for analysis, comparing the gene expression levels of *α-Sma*, *Il-6*, *Tgf-β1*, *Nf-κb*, and *Ctgf* in the posterior joint capsule tissue.

**Table 1:** Genes and primer sequences used for qPCR

Gene	Primer	Sequence
*Gapdh*	Forward	AACGACCCCTTCATTGACCT
	Reverse	CCCCATTTGATGTTAGCGGG
*α-Sma*	Forward	CATCATGCGTCTGGACTTGG
	Reverse	CCAGGGAAGAAGAGGAAGCA
*Il-6*	Forward	CCACCCACAACAGACCAGTA
	Reverse	ACTCCAGAAGACCAGAGCAG
*Tgf-β1*	Forward	CCCTACATTTGGAGCCTGGA
	Reverse	CGCACGATCATGTTGGACAA
N*f-κB*	Forward	AGAGGATGTGGGGTTTCAGG
	Reverse	GCTGAGCATGAAGGTGGATG
*Ctgf*	Forward	TCCCAAAATCTCCAAGCCTA
	Reverse	GTAATGGCAGGCACAGGTCT

### Statistical analysis

Statistical analysis was performed using GraphPad Prism 10.3.1 (GraphPad Software, San Diego, USA). Quantitative data are presented as bar charts, box plots with medians and quartiles, as mean values ± standard deviation, or as area under the curve (AUC) diagrams. The Mann–Whitney test was used to analyze joint contracture angles and analysis of variance (ANOVA) for gene expression. Gene expression comparisons and cell counts were made using one-way ANOVA (comparing ΔCt values or myofibroblasts). All measurements were conducted in triplicate, and statistical significance was defined as a *p*-value < 0.05. In the box plots, the whiskers extend to the minimum and maximum values, the line through the box indicates the median, and circles represent individual data points.

### Graphic illustrations

The schematic illustrations were created using the BioRender platform (https://bioRender.com).

## Results

### Complications and exclusions

During the 2-week observation period, there were no relevant abnormalities that led to the premature euthanasia of an animal. However, X-ray examination before implant removal confirmed a femur fracture proximal to the K-wire insertion in one animal in the placebo group. The surgically adjusted joint angle of 35° remained unchanged. Arthrometric measurement was not performed on the fractured leg. However, the posterior joint capsule was evaluated for qPCR and histology.

One sample from the control group intended for histopathological examination had to be excluded due to incomplete decalcification. Two samples intended for qPCR analysis were not stored in accordance with the protocol, which also led to their exclusion.

### Biomechanic evaluation

#### Assessment of the physiological extension deficit

The knee joint angle (JA) was defined as the angle formed by the intersection of the longitudinal axis of the femur and the line extending from the center of the tibial plateau to the upper ankle joint. Full geometric extension (fgE), represented by a 180° joint angle, is hindered by the posterior joint capsule and the posterior cruciate ligament. The difference between the fgE and the full physiological extension angle (pEA) was considered the physiological extension deficit (pED). In a healthy rat knee joint, application of a torque of 35 Nmm results in full physiological extension without causing damage to the capsular or ligamentous structures [7, 20]. The uninjured left knees of the placebo group were used to determine the pED. A torque of 35 Nmm results in an average extension of 150° (± 20.6°) in the healthy knee. Therefore, the pED (where pED = fgE − pEA) is calculated to be 30° (Fig. 4B).

#### Effect of nintedanib on biomechanics

##### Static evaluation of the joint contracture

In our previous studies, we observed that inhibition of one of the selected pro-fibrotic receptors did not result in significant attenuation of the onset of post-traumatic arthrofibrosis in our experimental model. [24, 26, 27]. Consequently, we posited that simultaneous inhibition of multiple pro-fibrotic signaling pathways, achievable through the multi-target inhibitor nintedanib, would quantifiably mitigate the progression of post-traumatic arthrofibrosis in our rat model [28].

To determine the antifibrotic effect of nintedanib on the biomechanical level, the restricted extension angle (rEA) of posterior capsule contracture was examined 2 weeks after trauma. At this point in time, arthrofibrosis had progressed largely in our model [26, 27]. The contracture angle (CA) was defined as the angle obtained by subtracting the rEA at 35 Nmm torque from pEA (CA = pEA − rEA) (Fig. 4B). Under the influence of nintedanib, a CA of 68.1 ± 12.6° developed compared to a CA of 84.8° ± 11.1° under PBO. On average, the difference in CA was 16.7°, with a corresponding increase in joint mobility in the nintedanib group (*p* < 0.01, Mann–Whitney test) (Fig. 5A).

##### Dynamic evaluation of the joint contracture

In addition, dynamic arthrometry was employed to determine the force required to move the JA from 60 to 180°. To quantify this, the area under the curve representing the force applied by the arthrometer and the corresponding extension angle was calculated. The applied force was determined by multiplying the amperage used by a device-specific force constant (1 N = 12 * 10 mA). Results indicated that extension of the knee joint from 60 to 80° required less force with nintedanib than with placebo (*p* ≤ 0.05, multiple *t*-test with Welch correction). Additionally, up to a knee extension angle of 90°, the nintedanib showed a trend towards superior efficacy (*p* = 0.07). However, beyond 90°, the measured values showed increasing variability, rendering the differences statistically insignificant (Fig. 5B).

### Pathohistological evaluation

Immunohistochemical staining was used to identify α-SMA positive ( +) myofibroblasts, allowing for their distinction from α-SMA( +) endothelial cells and smooth muscle cells in the vessel walls, as well as α-SMA negative( −) fibroblasts and proto-myofibroblasts [25]. Following the exclusion of vessel-associated cells, α-SMA( +) cells were normalized to the total cell count within the same histological section (Fig. 6). Analysis of the posterior capsule sections revealed that nintedanib treatment significantly reduced the relative number of myofibroblasts at week 2 compared to the placebo group (10.3 ± 7.8% vs. 24.5 ± 6.7%, *p* ≤ 0.01). In contrast, sections from the control group showed almost no myofibroblasts. There was also a significant difference between the nintedanib group and the control group. However, this was less prominent (10.3 ± 7.8% vs. 1.1 ± 0.78%, *p* ≤ 0.05, one-way ANOVA and post hoc Tukey multiple comparison test). The total number of cells in the nintedanib group (118 ± 34 cells/HPF) was similar to that in both the placebo (83 ± 47 cells/HPF) and control (78 ± 38 cells/HPF) groups. There was a significant reduction in myofibroblasts in the control group compared to the placebo group (control: 1 ± 1 vs. placebo: 20 ± 12 cells/HPF, *p* < 0.01, one-way ANOVA and post hoc Tukey multiple comparison test). There was also a tendency for myofibroblasts to be reduced in the nintedanib group (12 ± 9 cells/HPF). However, this difference was not significant compared to either the control or placebo groups.

### Molecular pathology of the posterior joint capsule

**Fig. 7:**
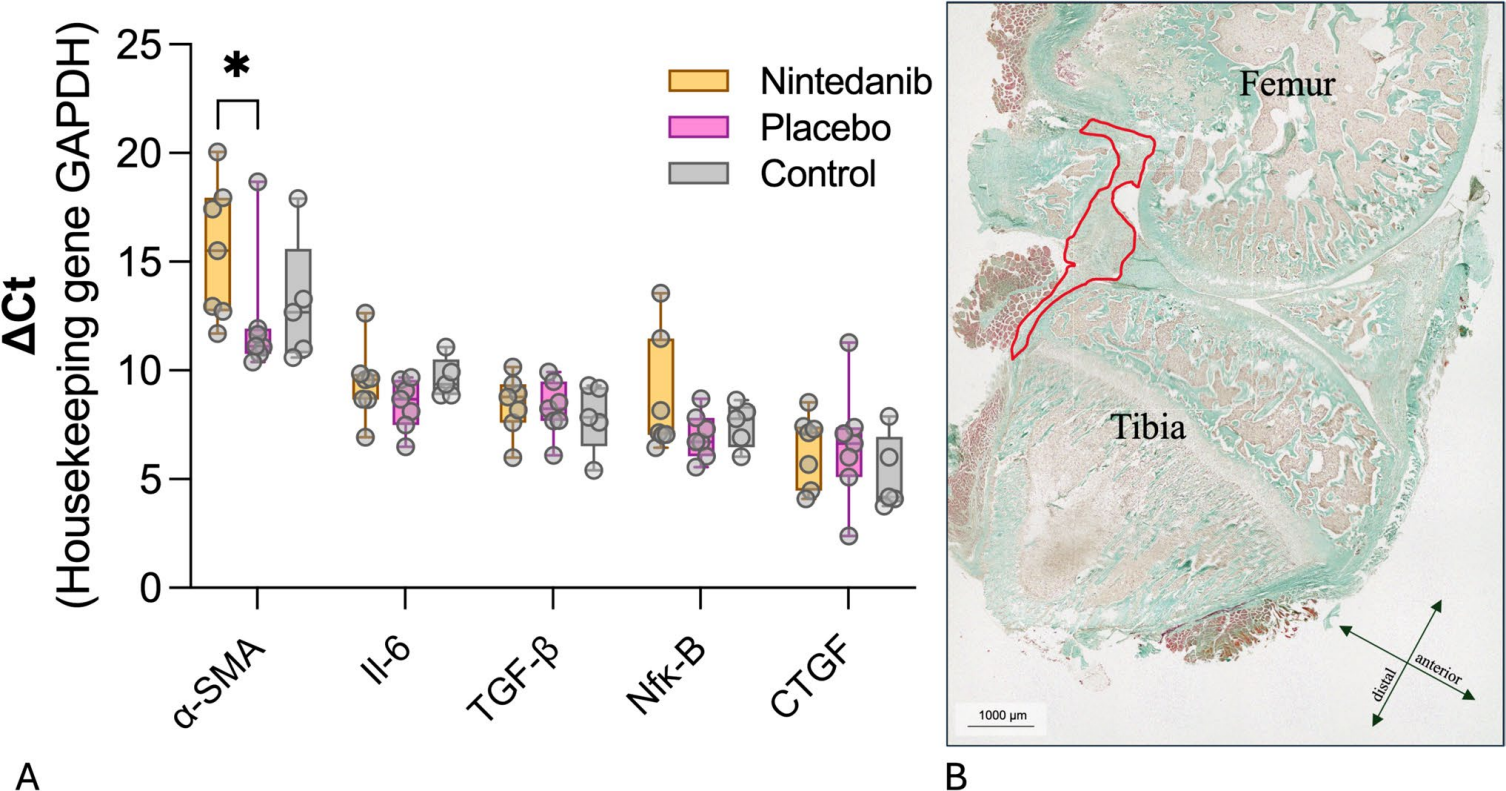
Profibrotic gene expression. **A** The diagram shows the gene expression (ΔCt) of *α-Sma*, *Il-6*, *Tgf-β*, *Nf-κb*, and *Ctgf*, with housekeeping gene *Gapdh*. Statistically significant differences between groups are marked with ** (*p* ≤ 0.05, one-way ANOVA, post hoc Tukey multiple comparison test). **B** Sagittal section through the knee joint (Masson–Goldner stain). The posterior joint capsule, outlined in red, was analyzed for profibrotic gene expression

The expression levels of profibrotic genes and their surrogates were examined in the posterior joint capsule 2 weeks after trauma (Fig. 7). Ct values were normalized to *Gapdh*. Two weeks after trauma, all three groups showed nearly identical expression levels of the genes *Il*-6, *Tgf-β*, *Nf-κb*, and *Ctgf*. *α-Sma* served as a marker of myofibroblast differentiation [29]. Compared to placebo, nintedanib reduced the expression of *α-Sma* more than 11-fold (ΔCt 15.42 ± 3.14 vs. 11.95 ± 2.89, p < 0.05 one-way ANOVA and post hoc Tukey multiple comparison test). No significant difference in *α-Sma* expression levels was found between the placebo and control groups.

## Discussion

A growing understanding of both common and organ-specific mechanisms underlying the pathophysiology of fibrotic diseases has enabled the development of the highly effective small molecule tyrosine kinase inhibitor, nintedanib [30, 31]. Nintedanib has become a key agent in the treatment of various forms of pulmonary fibrosis, including interstitial lung diseases (ILDs), which are among the most common fibrotic extra-articular manifestations of rheumatoid arthritis. This association suggests a particularly close pathophysiological relationship not only between disease patterns, but also between lung and joint tissues [13, 32]. PTJC, another fibrotic condition that exhibits significant genetic and functional ontological similarities to rheumatoid arthritis, currently lacks any potent pharmacological alternative [13, 33]. Controlling abnormal signaling cascades could prove more effective than current therapeutic standards, that rely on manual interventions, by avoiding further traumatization of the joint.

This study successfully demonstrates the beneficial effects of orally administered nintedanib on the formation of PTJC in surgically traumatized knee joints of 26 male Sprague–Dawley rats over a 2-week period.

The duration of immobilization, and therefore the length of time for which the drug intervention was required, was based on our previous research into traumatic arthrofibrosis, and on the anticipated measurable antifibrotic effect of nintedanib. In our model, the acute pro-inflammatory phase was fully resolved 2 weeks after trauma. It can therefore be assumed that the joint contracture was almost completely established by this time [24, 26, 27]. Our gene expression analysis of the posterior joint capsule confirms this. At this stage, the mediators of the pro-inflammatory phase had already subsided. As a result, the expression levels of *Il-6, Nf-κb, Tgf-β*, and *Ctgf* were comparable in the placebo and control groups. These results are in line with the work on the molecular landscape of post-traumatic arthrofibrosis in the rabbit model by Morrey et al. Microarray analysis of the temporal expression of 380 genes revealed that pro-inflammatory gene expression was upregulated only a few hours after trauma and normalized by the end of the second week [34].

The increased capsular contracture observed, compared to our previous studies, is likely attributed to the additional intercondylar bone lesion and concomitant hemarthrosis. Joint bleeding has been shown in various animal models and clinical studies to cause early-onset synovitis with persistent infiltration of the tissue by myeloid cells [35–37]. The induced hemarthrosis in our model likely acts as a catalyst for the development of arthrofibrosis.

In the absence of comparative data on PTJC or other forms of arthrofibrosis in animal models, the optimal dose and duration of nintedanib remain to be established. The agent, however, is also expected to have a measurable antifibrotic effect over a course of 14 days, as this effect has been demonstrated after the same time period in a variety of rat models, including the lung and urinary tract [38–40]. The dose of 5 mg/kg twice daily p.o. was chosen based on the NOAEL in rats reported in the literature for a 14-day intervention period. During this period, an antifibrotic effect could still be demonstrated in a bleomycin-induced pulmonary fibrosis model in the rat [15, 18, 38].

Our research indicates that therapy with nintedanib is advantageous at all levels of our PTJC model. However, despite the prophylactic administration of the drug, the development of contracture could not be completely prevented. A biomechanical improvement of the contracture of 16.7° under the influence of nintedanib could nevertheless hold significant clinical importance, since even mild flexion contractures in the knee joint (< 15°) are linked to a high degree of disability [41]. A significant decrease in the number of myofibroblasts indicates nintedanib’s anti-fibrotic footprint at the cellular level. In addition, the reduced *α-Sma* expression levels, which are considered a hallmark of the fibroblast-to-myofibroblast transition, suggest that nintedanib attenuates fibrotic remodeling of the posterior joint capsule [42, 43].

The incomplete effect of the tyrosine kinase inhibitor on the PTJC could be attributable to the following factors. Considering the reduced bioavailability of the orally administered nintedanib, it is most likely that the selected dose exerted only a partial antifibrotic effect on the joint. Wollin et al. also came to this conclusion, demonstrating an incomplete antifibrotic effect of nintedanib at the same dosage in their model of bleomycin-induced pulmonary fibrosis in rats. Full inhibition of pulmonary fibrosis was only achieved with a prophylactic dosage of ≥ 30 mg/kg/day [15]. In addition to a higher dosage, the bioavailability could be significantly improved by local application, similar to the aerosol administration of nintedanib in pulmonary fibrosis [44].

A potential drug-drug interaction in our experimental design that would have affected the metabolism of the tyrosine kinase inhibitor appears to play a subordinate role. In contrast to tramadol, which was administered concomitantly for the initial 4 postoperative days, nintedanib is metabolized predominantly by UDP-glycosyltransferase independently of Cytochrome P450 (CYP) enzymes [15, 45].

As mentioned above, Morrey et al. examined the temporal sequence of more than 380 genes involved in post-traumatic inflammation in their rabbit model of PTJC, over a 14-day period. Utilizing microarray data and qPCR analyses of the posterior joint capsule, it was determined that the inflammatory response was completed after 14 days and that the greatest inflammatory changes occurred within the first 24 h. Pro-inflammatory IL-1 expression, an IL-6 inductor, exhibited a peak at 6 h and returned to baseline levels by 72 h post-trauma [34]. As the peak plasma concentrations of nintedanib were described after approximately 5.5 h in rats, it is conceivable that the acute phase of inflammation was not fully addressed by the tyrosine kinase inhibitor [46]. One plausible explanation for this phenomenon is the presence of “no-return points” in the early phase of fibrosis that had already been crossed.

A direct comparison of the efficacy of nintedanib with previous pharmacological studies is significantly limited due to high levels of heterogeneity across animal studies, as outlined by Palacios-Díaz et al. [47]. Differences in species, trauma models, administration of the agent (route, dosage, and duration), immobilization protocols, and outcome assessment methods (e.g., torque thresholds and re-mobilization) make cross-study efficacy comparisons unreliable. Nevertheless, nintedanib—along with ketotifen, montelukast, and members of the corticosteroid and NSAID families—is one of the few agents whose systemic effect has a biomechanically measurable effect on joint contracture [7–10].

## Conclusion

In conclusion, nintedanib has confirmed its antifibrotic efficacy for the first time in the development of post-traumatic joint contracture. It is one of the few drugs with a measurable impact on this condition. Although the development of PTJC was not completely prevented, oral administration of a relatively low dose of the drug significantly enhanced joint mobility in arthrofibrotic knee joints over a 2-week period. This partial effect was also evidenced by the notable reduction of myofibroblasts and the diminished transition of fibroblasts to myofibroblasts in the posterior joint capsule.

Although nintedanib was not able to completely prevent PTJC in our model, inhibition of multiple signaling cascades involved in arthrofibrosis represents a promising strategy for developing a targeted pharmacotherapy for PTJC.

## Data Availability

The datasets generated and analyzed during the current study are available from the corresponding author on reasonable request.

## Abbreviations

*a-Sma*: Alpha-smooth muscle actin
BID: Bis in die
CA: Contracture angle
CD34: Cluster of differentiation 34
cDNA: Complementary deoxyribonucleic acid
CTGF / -R: Connective tissue growth factor / -receptor
DKK3-SLF: Dickkopf 3 sublining fibroblasts
EDTA: Ethylenediaminetetraacetic acid
FGF / -R: Fibroblast growth factors / -receptor
FMT: Fibroblast-to-myofibroblast transition
*Gapdh*: Glyceraldehyde-3-phosphate dehydrogenase
H&E: Hematoxylin and eosin stain
*Il-6*: Interleukin 6
ILD: Interstitial lung disease
JA: Joint angle
K-wire: Kirschner wire
mRNA: Messenger ribonucleic acid
*Nf-κb*: Nuclear factor ‘kappa-light-chain-enhancer’ of activated B-cells
NOAL: No-observed-adverse-effect level
rEA: Restricted extention angle
PDGF / -R: Platelet-derived growth factor / -receptor
pEA: Physiological extension angle
pED: Physiological extension deficit
PTJC: Post traumatic joint contracture
qPCR: Quantitative polymerase chain reaction
RTK: Receptor tyrosine kinases
*Tgf-β1*: Transforming growth factor beta1
TRIS: Tris(hydroxymethyl)aminomethane
